# Functional characterization of two DYRK1B variants causative of AOMS3

**DOI:** 10.1186/s13023-024-03183-0

**Published:** 2024-06-12

**Authors:** Silvia Detro-Dassen, Anna Sternberg, Sonja Maria Lehmann, Katharina Schwandt, Stefan Düsterhöft, Walter Becker

**Affiliations:** 1 Institute of Pharmacology and Toxicology, RWTH Aachen University, Aachen, Germany; 2 Institute of Molecular and Cellular Anatomy, RWTH Aachen University, Aachen, Germany; 3 Institute of Molecular Pharmacology, RWTH Aachen University, Aachen, Germany

## Abstract

**Background:**

Two new missense variants (K68Q and R252H) of the protein kinase DYRK1B were recently reported to cause a monogenetic form of metabolic syndrome with autosomal dominant inheritance (AOMS3).

**Results:**

Our in vitro functional analysis reveals that neither of these substitutions eliminates or enhances the catalytic activity of DYRK1B. DYRK1B-K68Q displays reduced nuclear translocation.

**Conclusion:**

The pathogenicity of DYRK1B variants does not necessarily correlate with the gain or loss of catalytic activity, but can be due to altered non-enzymatic characteristics such as subcellular localization.

**Supplementary Information:**

The online version contains supplementary material available at 10.1186/s13023-024-03183-0.

## Dear editor

We read with great interest the recent article entitled “Two novel variants in DYRK1B causative of AOMS3: expanding the clinical spectrum” by Mendoza-Caamal et al. [1]. Their identification of two novel DYRK1B missense mutations (K68Q and R252H) causative of abdominal obesity metabolic syndrome-3 (AOMS3) provides a long-awaited follow-up to the original discovery of AOMS3 [2]. AOMS3 is a rare form of metabolic syndrome with monogenetic inheritance that is caused by heterozygous mutations of the DYRK1B gene. Two missense mutations (R102C and H90P) in DYRK1B were originally discovered to cause AOMS3 [2]. While the R102C allel was reported to show gain-of-function activities in cell culture assays [2], experiments from our lab showed no effect of either variant on the catalytic activity of DYRK1B in biochemical assays. Rather, both mutations compromised the conformational stability of the catalytic domain [3].

**Fig. 1:**
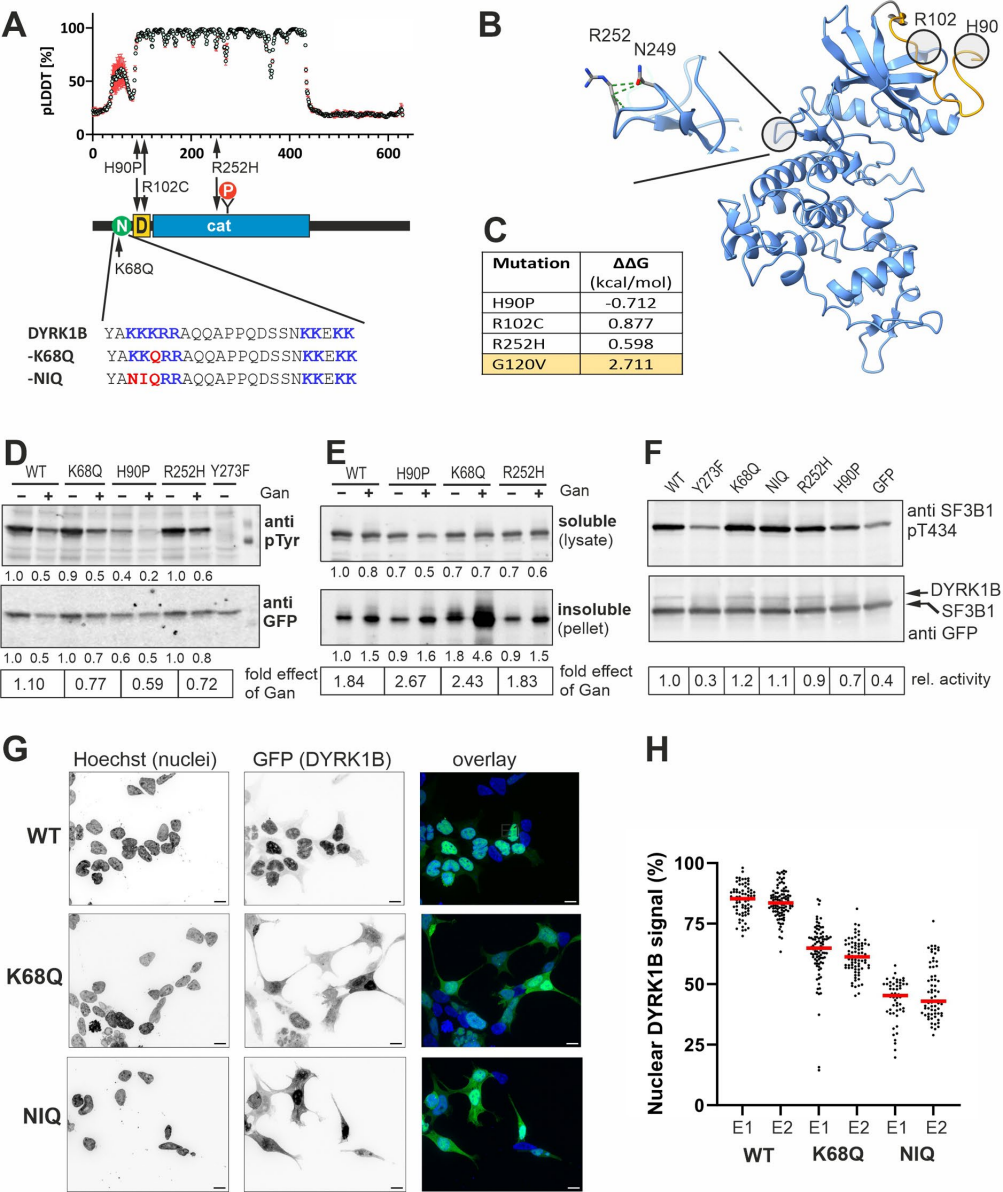
Analysis of DYRK1B point mutants. **A**, Mapping of AOMS3 missense mutations to the structure of DYRK1B. Structural features include the bipartite nuclear localization sequence (N) and the DYRK homology box (D). The autophosphorylated Tyr273 in the catalytic domain (cat) is indicated (PY). The graph shows the predicted local distance difference test (pLDDT) scores with standard deviation per position in the five structural models generated by AlphaFold2. The sequence alignment illustrates the K68Q substitution and the NIQ mutant (K66N, K67I, K68Q) that was generated as a control to disrupt the left part of the NTS (panel H). **B**, The AlphaFold model of the structured region of DYRK1B (DH box plus catalytic domain, amino acids 89–431) was subjected to molecular dynamics simulation (GROMACS2020.6: AMBER99SB-ILDN force field; neutralized system with 150 NaCl in water; 300 K and 1 atm using the v-rescale temperature and Parrinello − Rahman pressure coupling method during production). RMSD equilibrium was reached after 10 ns. Frame at 90 ns of the molecular dynamics simulation was used for illustration and for calculating ΔΔG values of mutations using the FoldX algorithm. The enlarged cutout illustrates the van der Waals contacts of R252 with N249. **C**, Mean ΔΔG values for the pathogenic mutations in the structured region of DYRK1B as detailed in supplementary Fig. S2. For comparison, ΔΔG was also calculated for the catalytically inactive G120V substitution [5]. **D-F**, HEKtsa201 cells were seeded in 6-well plates (250,000 cells per well) and transiently transfected with expression plasmids for GFP-DYRK1B constructs as indicated. Ganetespib (Gan, 100 nM) was added for 24 h before cell lysis. Two days after transfection, cells were either lysed on ice in native lysis buffer containing a non-denaturing detergent (**D** and **E**, 1.0% Igepal CA 630) or by denaturing lysis at 96 °C (**F**, 1% SDS). In **D**, tyrosine autophosphorylation of DYRK1B in the soluble fraction was detected with the help of a phospho-HIPK2 antibody (Thermo Fisher Scientific, #PA5-13045; RRID: AB_10987115) as previously described [3]. DYRK1B-Y273F serves as a technical control for antibody specificity. The effect of ganetespib on the relative phosphotyrosine content (pTyr/GFP) is indicated. To determine the relative proportion of insoluble GFP-DYRK1B (**E**), soluble and insoluble fractions were separated by centrifugation (5 min, 14,000 rpm). The effect of ganetespib on the proportion of insoluble GFP immunoreactive material is indicated. **F**, Cellular DYRK1B activity was assessed by detecting the phosphorylation of SF3B1 at T434 in cells that had been co-transfected with expression plasmids for GFP-SF3B1 (500 ng/well) and the indicated GFP-DYRK1B constructs (50 ng/well). Background phosphoT434 levels in GFP transfected cells are due to endogenous DYRK1 activity. **G-H**, Subcellular localization of GFP-DYRK1B constructs in transiently transfected HEK293-tsa201 cells was detected by fluorescence microscopy (Zeiss ApoTome.2). Cells were fixed with paraformaldehyde, permeabilized with 0.2% Triton-X, and nuclei were stained with Hoechst 33,342. Representative images from two independent experiments (E1, E2) are shown in **G** (scale bar, 10 μm). The scatter plot (**H**) shows the proportion of nuclear GFP fluorescence as quantitated with the help of ImageJ/FIJI for at last 50 randomly selected cells per experimental condition

The novel DYRK1B missense mutations offer new opportunities to gain further insight into the role of DYRK1B in the pathogenesis of AOMS3. K68 is a highly conserved lysine residue in the putative nuclear targeting sequence (NTS) of DYRK1B (Fig. 1A). R252 is located in the catalytic domain and is conserved in the DYRK family, except for DYRK4. However, this residue is neither directly involved in catalysis nor does it belong to the motifs that coordinate the kinase domain to create an active conformation [4]. Our in silico structural analyses suggest that R252 undergoes interactions that may provide a small contribution to the local conformational stability in the catalytic domain (Fig. 1B and C). However, the effect of the R252H mutation is much weaker than that of the G120V substitution, which was recently identified as loss-of-function mutation [5]. We have experimentally characterized the new DYRK1B missense mutations to address the key question whether AOMS3 is caused by gain or loss of function of the DYRK1B protein.

Guided by our previous analysis of pathogenic variants of DYRK1A and DYRK1B [3, 6] we first assessed the phosphotyrosine content of the DYRK1B missense variants. Autophosphorylation of a specific tyrosine residue in the activation loop (Y273 in DYRK1B) occurs during the maturation of DYRKs and serves as a marker for the correct folding and conformational integrity of the catalytic domain. The K68Q and R252H substitutions did not affect tyrosine autophosphorylation of DYRK1B, indicating that both variants are catalytically active protein kinases (Fig. 1D).

Maturation of DYRK1B is assisted by the HSP90 chaperone system [3]. We have previously shown that the HSP90 inhibitor ganetespib impairs tyrosine autophosphorylation of DYRK1B and induces aggregation of the protein. Ganetespib had a marginal effect on the phosphotyrosine content of K68Q and R252H, if any (Fig. 1D). DYRK1B-R252H did not differ from wild type DYRK1B with regard to the accumulation in insoluble aggregates (Fig. 1E), while DYRK1B-K68Q resembled DYRK1B-H90P in its increased sensitivity to the effect of ganetespib [3] (Fig. 1E).

Next we asked whether the new variants might compromise the catalytic activity of DYRK1B towards a substrate protein. Splicing factor 3B1 (SF3B1) is a substrate of DYRK1B, and the level of phosphorylated T434 is a well-established marker of DYRK1 activity in cell-based assays [7]. No difference between wild type DYRK1B and the K68Q or R252H variants was observed in this assay (Fig. 1F). In addition, DYRK1B-K68Q or R252H were not impaired in their capacity of binding DCAF7 (supplementary Fig. S3), which acts as a substrate-recruiting adaptor protein [8].

We finally asked whether K68Q affected the subcellular localization of GFP-DYRK1B, as proposed by Mendoza-Caamal et al. [1]. About 80% of wild type GFP-DYRK1B but only 60% of DYRK1B-K68Q was localized in the nucleus of transiently transfected HEK293 cells (Fig. 1G and H). This result indicates that the nuclear import of DYRK1B is impaired but not prevented by the missense mutation in the presumed nuclear targeting sequence (NTS). Indeed, nuclear import was still possible when three of the lysine residues in the NTS were mutated.

Taken together, the new DYRK1B variants cause neither loss- nor gain-of-function effects regarding their enzymatic properties, as evaluated through tyrosine autophosphorylation and catalytic activity towards the downstream substrate SF3B1. These results align with prior observations [3] that the pathogenicity of DYRK1B variants is not strictly linked to the loss of enzymatic activity. Of note, this analysis aimed at identifying blatant changes in protein function and does not exclude the existence of more subtle defects in specific downstream pathways. The significance of the increased dependence on HSP90 chaperoning in the aggregation assay remains unclear, given the normal function of K68Q in the SF3B1 assay. In contrast, the impaired nuclear import of DYRK1B-K68Q may result in a gradual loss of the pertinent nuclear functions of DYRK1B.

After completion of our investigations, Folon and co-workers [5] reported the characterization of new DYRK1B variants. The authors identified 6 total loss-of-function DYRK1B mutants that were associated with monogenic obesity and hyperglycemia, including a nonsense mutation in the catalytic domain. Another pathogenic *DYRK1B* variant was identified in a patient with a familial form of metabolic syndrome and severe neurodevelopmental abnormalities [9]. This allel contains an intronic point mutation that disrupts a splice donor site, and the authors speculate that the more complex syndrome in the affected family members compared to other AOMS3 patients might be due to haploinsuffiency [9]. In aggregate, current evidence argues against the hypothesis that AOMS3 is caused by gain-of-function DYRK1B variants, although non-catalytic effects cannot be excluded. Hopefully, the discovery of further *DYRK1B* mutations will foster more functional studies on the role of this protein kinase in the pathogenesis of metabolic syndrome.

### Electronic supplementary material

Below is the link to the electronic supplementary material.

Supplementary Figures (S1, S2, S3)

Supplementary Material 2 (Raw data to Fig. 1H)

## Data Availability

The datasets supporting the conclusions of this article are included within the article and its additional file. The expression plasmids are available on reasonable request.
