# Supplementary material for: Functional characterization of two DYRK1B variants causative of AOMS3
Source: Orphanet J Rare Dis. 2024 Jun 12;19:233. doi: 10.1186/s13023-024-03183-0 (PMC11167895; doi:10.1186/s13023-024-03183-0)
Supplement: Supplementary file 1 — Supplementary Figures (S1, S2, S3) [file 13023_2024_3183_MOESM1_ESM.pdf]

Supplementary data to

Letter to the editor:

Functional characterization of two DYRK1B variants causative of AOMS3

by Silvia Detro-Dassen, Anna Sternberg, Sonja Maria Lehmann, Katharina Schwandt,

Stefan Düsterhöft, Walter Becker

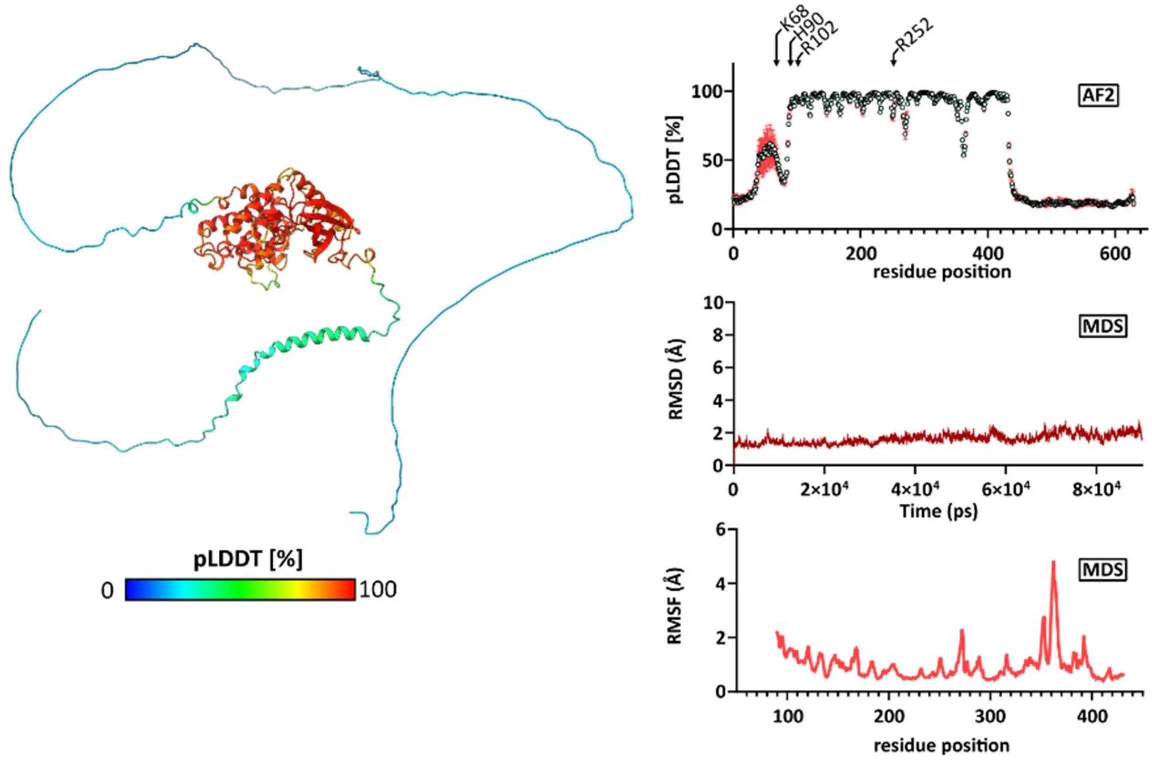

Fig. S1 Molecular Dynamics Simulation of DYRK1B (additional data to Fig. 1A-B)

The structural model of DYRK1B is stained to illustrate the predicted local distance difference test (pLDDT) scores in five models generated by AlphaFold2 (AF2). High confidence values (>90) are limited to the catalytic domain while the N-terminal and C-terminal domains show low confidence values (<50). The lower two graphs show the results of the molecular dynamics simulation (MDS) for the structured region of DYRK1B (DH box plus catalytic domain, amino acids 89-431). Root mean square deviation (RMSD) equilibrium was reached within 10 ns. The per residue Root Mean Square Fluctuation (RMSF) scores show the deviation of the position of the atomic positions with respect to a reference position over time and serve as measure of random motion. The peak around 350-370 reflects the conformational instability of the CMGC loop.

	Mean ΔΔG (kcal/mol)
H90P	-0.71194
R102C	0.877457
G120V	2.711459
H179L	-0.54542
R252H	0.597891
D259N	-0.5789
P282L	1.308252

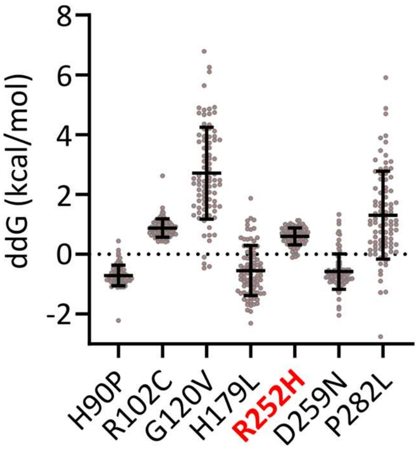

Fig. S2 Mean ΔΔG values for DYRK1B pathogenic variants (additional data to Fig. 1C)

The table lists pathogenic DYRK1B missense variants that affect the catalytic domain or DH box (Refs 1,2 5 in the manuscript). To perform a thorough analysis of the potential effects of mutations in DYRK1B, a total of 85 conformations/frames were selected from the molecular dynamics (MD) simulation (right panel). These selected conformations were derived from the last 15 nanoseconds of the simulation, within the root-mean-square deviation (RMSD) equilibrium state. For each of these conformations, a computational mutagenesis process was applied to the specified mutations using the FoldX algorithm. The change in stability induced by mutagenesis in each conformation/frame was quantified. The mean and standard deviation of the change in Gibbs free energy (ΔΔG) were then calculated for all the conformations that underwent mutagenesis (left table).

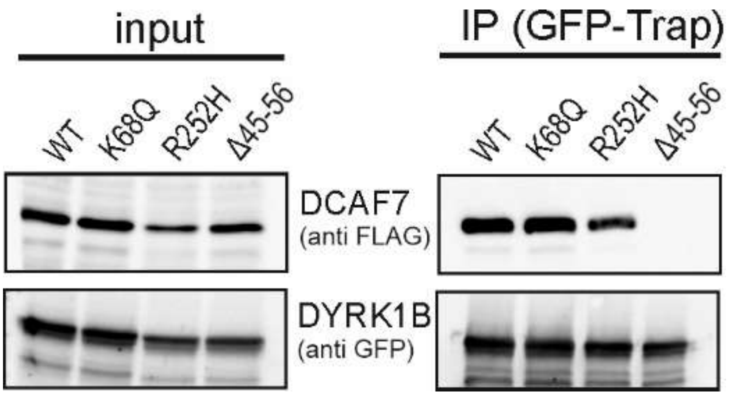

Fig. S3 Co-immunoprecipitation of DCAF7 and DYRK1B variants

HEKtsa201 cells were transiently co-transfected with expression plasmids for FLAG-DCAF7 (Glenewinkel et al. 2016) and the indicated GFP-DYRK1B constructs. As a negative control, we constructed a deletion mutant (Δ45-56) that was designed analogously to the DCAF7-nonbinding mutant DYRK1A-Δ93-104 (Glenewinkel et al. 2016). Two days after transfection, cells were lysed on ice in native lysis buffer, cell lysates were subjected to immunoprecipitation using GFP-trap magnetic beads (Chromotek, RRID AB_2631357), and bound proteins were detected by immunoblotting with antibodies directed against the FLAG or GFP tag as indicated (anti DCAF7, Abcam mAb EPR8712; anti GFP, Rockland RRID:AB_218182). Aliquots of the whole cell lysates are shown as input controls.

Glenewinkel F, Cohen MJ, King CR, Kaspar S, Bamberg-Lemper S, Mymryk JS, Becker W. The adaptor protein DCAF7 mediates the interaction of the adenovirus E1A oncoprotein with the protein kinases DYRK1A and HIPK2. Sci Rep. 2016 6:28241.

uncropped blots of the experiment shown in Fig. 1D (Exp.20220325)

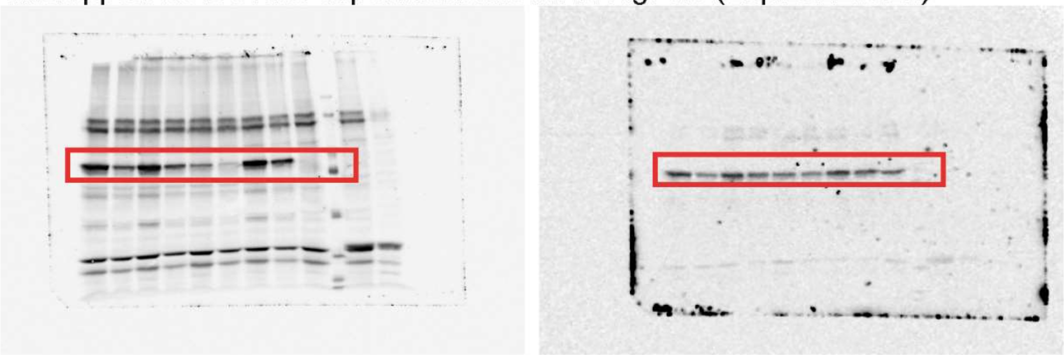

uncropped blots of the experiment shown in Fig. 1E (Exp.20220210)

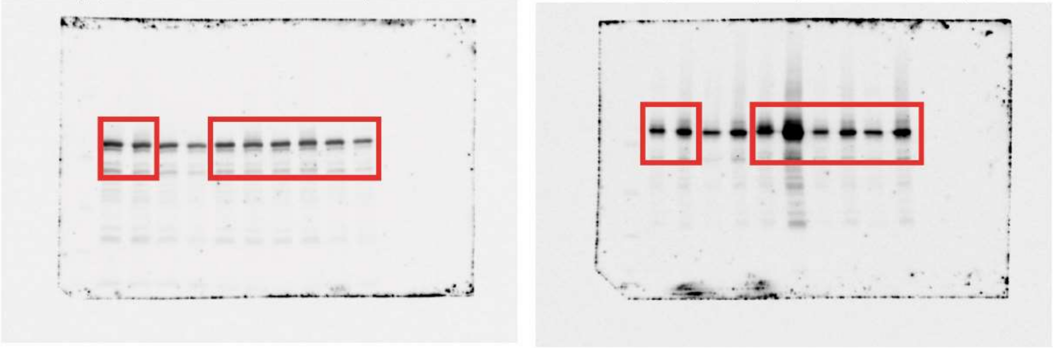

uncropped blots of the experiment shown in Fig. 1F (Exp.20220718)

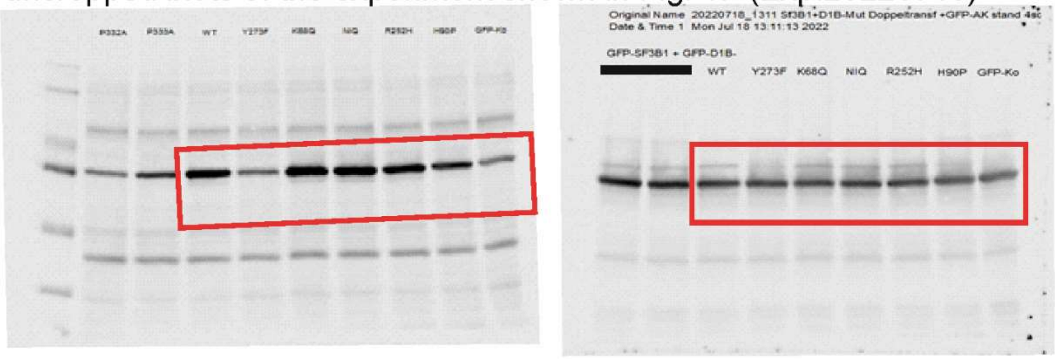

uncropped blots of the experiment shown in Fig. S2 (Exp.20240207)

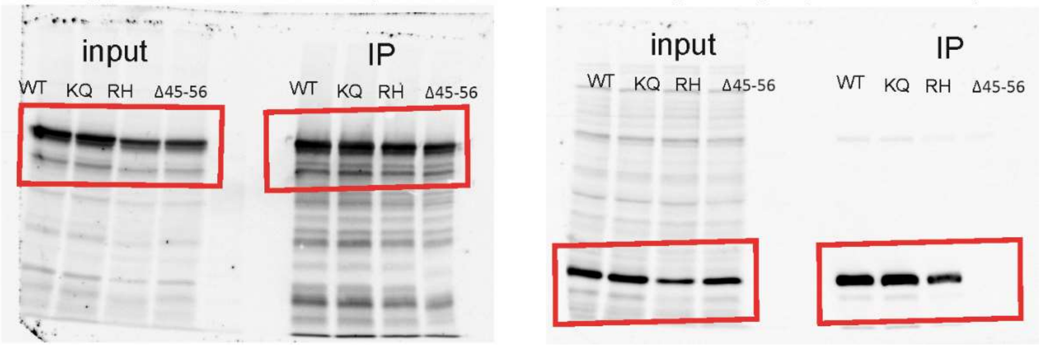

Fig. S4 Uncropped Western blots provided as additional data to Fig. 1D-F and Fig. S3
